# Eating disorder hospitalizations among children and youth in Canada from 2010 to 2022: a population-based surveillance study using administrative data

**DOI:** 10.1186/s40337-023-00957-y

**Published:** 2024-01-02

**Authors:** Stephanie Toigo, Debra K. Katzman, Ellie Vyver, Steven R. McFaull, Ithayavani Iynkkaran, Wendy Thompson

**Affiliations:** 1 Centre for Surveillance and Applied Research, Public Health Agency of Canada, 785 Carling Avenue, Ottawa, ON K1A 0K9 Canada; 2 Division of Adolescent Medicine, Department of Pediatrics, The Hospital for Sick Children and University of Toronto, Toronto, Canada; 3 Section of Adolescent Medicine, Department of Pediatrics, Alberta Children’s Hospital and University of Calgary, Calgary, Canada

## Abstract

**Background:**

Eating disorders (EDs) are severe mental illnesses associated with significant morbidity and mortality. EDs are more prevalent among females and adolescents. Limited research has investigated Canadian trends of ED hospitalizations prior to the COVID-19 pandemic, however during the pandemic, rates of ED hospitalizations have increased. This study examined rates of ED hospitalizations among children and youth in Canada from 2010 to 2022, by sex, age, province/territory, length of stay, discharge disposition and ED diagnosis.

**Methods:**

Cases of ED hospitalizations among children and youth, ages 5 to 17 years, were identified using available ICD-10 codes in the Discharge Abstract Database from the 2010/11 to 2022/23 fiscal years. The EDs examined in this study were anorexia nervosa (F50.0), atypical anorexia nervosa (F50.1), bulimia nervosa (F50.2), other EDs (F50.3, F50.8) and unspecified EDs (F50.9). Both cases of total and first-time ED hospitalizations were examined. Descriptive statistics and trend analyses were performed.

**Results:**

Between 2010/11 and 2022/23, 18,740 children and youth were hospitalized for an ED, 65.9% of which were first-time hospitalizations. The most frequent diagnosis was anorexia nervosa (51.3%). Females had significantly higher rates of ED hospitalization compared to males (66.7/100,000 vs. 5.9/100,000). Youth had significantly higher rates compared to children. The average age of ED hospitalization was 14.7 years. Rates of ED hospitalizations were relatively stable pre-pandemic, however during the pandemic (2020–2021), rates increased.

**Interpretation:**

Rates of pediatric ED hospitalizations in Canada increased significantly during the pandemic, suggesting that there may have been limited access to alternative care for EDs or that ED cases became more severe and required hospitalization. This emphasizes the need for continued surveillance to monitor how rates of ED hospitalizations evolve post-pandemic.

**Supplementary Information:**

The online version contains supplementary material available at 10.1186/s40337-023-00957-y.

## Abstract (plain language summary)

Eating disorders disproportionally affect children and youth, however, literature investigating long-term trends of eating disorder hospitalizations among children and youth in Canada is limited. We conducted a retrospective surveillance study, examining eating disorder hospitalizations among children and youth in Canada, from 2010 to 2022, by sex, age group, geography and eating disorder diagnosis. More than half of eating disorder hospitalizations examined during our study period were first-time hospitalizations. The most common eating disorder diagnoses were anorexia nervosa, followed by unspecified eating disorders. Youth had higher rates of eating disorders compared to younger children and females had higher rates compared to males. In Canada, rates of pediatric eating disorder hospitalizations increased during the pandemic. These results emphasize the need for continued surveillance to monitor how ED hospitalizations evolve post-pandemic, as well as prioritizing early intervention and treatment to help reduce the number of children and youth requiring hospitalization.

**Supplementary Information:**

The online version contains supplementary material available at 10.1186/s40337-023-00957-y.

## Background

Eating disorders (EDs) are life-threatening mental illnesses with some of the highest mortality rates among psychiatric disorders [1–4]. EDs often co-occur with other psychiatric or medical disorders, adding to treatment complexity [5]. Generally, EDs are characterized by disturbances in eating behaviours and preoccupation with body appearance and weight; however, these concerns vary by gender, age and ED diagnosis [2, 6]. EDs can occur across gender, age and sociodemographic groups. [2, 6, 7]

EDs are more prevalent among females [6, 8, 9]. Research estimates that the lifetime prevalence of anorexia nervosa (AN) is up to 4% in females and 0.3% in males, whereas the prevalence of bulimia nervosa (BN) can be up to 3% in females and 1% in males [7, 10]. Surveillance studies indicate that the prevalence of EDs in younger children (less than 11 years) is low, however estimates vary [11, 12]. The age of onset differs across EDs; AN is more frequent in adolescence (16–19 years) and rarely diagnosed after the age of 30 [10, 11, 13–15]. However, the age of onset of AN has decreased during the past decade, with some of the youngest reported cases now seen among children under the age of 10 [2, 8, 13–15]. The prevalence of BN onset is higher in young adults (20–29 years), with more cases continuing to be diagnosed into adulthood [14]. EDs can be difficult to treat, especially if not identified early and treated within the first three years of symptom onset [2, 16, 17].

Recent research has shown that the COVID-19 pandemic has contributed to an increase in ED hospitalizations among children and youth [17–21]. This increase may have resulted from changes in everyday routine, online schooling, social isolation and loneliness, increased social media use and disruptions to regular medical care [17, 22, 23].

Although there is research examining EDs in the pediatric population in Canada during the COVID-19 pandemic, there is minimal research examining long-term trends of ED hospitalizations. Ongoing surveillance of EDs is needed to identify changes in the prevalence and characteristics of existing and new patients requiring hospitalization. This can help inform future resource needs in hospitals as well as prioritize alternative forms of care that patients can access. Lastly, understanding how trends in ED hospitalizations changed during the pandemic compared to pre-pandemic can aid in future pandemic planning and post-pandemic recovery [17]. The objective of this study was to examine rates of ED hospitalizations among children and youth in Canada by age, sex, year, geography, length of stay, discharge disposition and ED diagnosis from 2010 to 2022.

## Methods

### Data sources and case selection

This work utilized the Discharge Abstract Database (DAD), a national administrative database managed by the Canadian Institute for Health Information (CIHI), which collects clinical and administrative information on hospital separations/discharges from all provinces and territories, excluding Quebec. This study did not include Quebec due to the Public Health Agency of Canada’s (PHAC) data sharing agreements with CIHI. This study was based on de‐identified data and conducted under PHAC’s surveillance mandate, therefore no ethics approval from an Institutional Review Board was necessary. The results of this study are reported in accordance with the Reporting of studies Conducted using Observational Routinely-collected Data guidelines [24].

This study analyzed acute care inpatient hospital discharge records. Uncertain and secondary ED diagnoses were excluded. These are diagnoses where there may have been some uncertainty and an exact diagnosis could not be provided or when the diagnosis was deemed non-significant to the episode of care.

The DAD uses the International Statistical Classification of Diseases and Related Health Problems, 10th revision, Canada (ICD-10) to classify diagnoses [25–27]. ED diagnoses were categorized with the following ICD-10 codes: AN (F50.0), atypical anorexia nervosa (AAN) (F50.1), BN (F50.2), other EDs (F50.3 and F50.8) and unspecified EDs (F50.9). The F50.8 code includes binge eating disorder, avoidant/restrictive food intake disorder (ARFID) and other EDs, however, we were unable to parse out these diagnoses from the F50.8 code as medical institutions in Canada do not specify the exact diagnosis in this category.

**Fig. 1:**
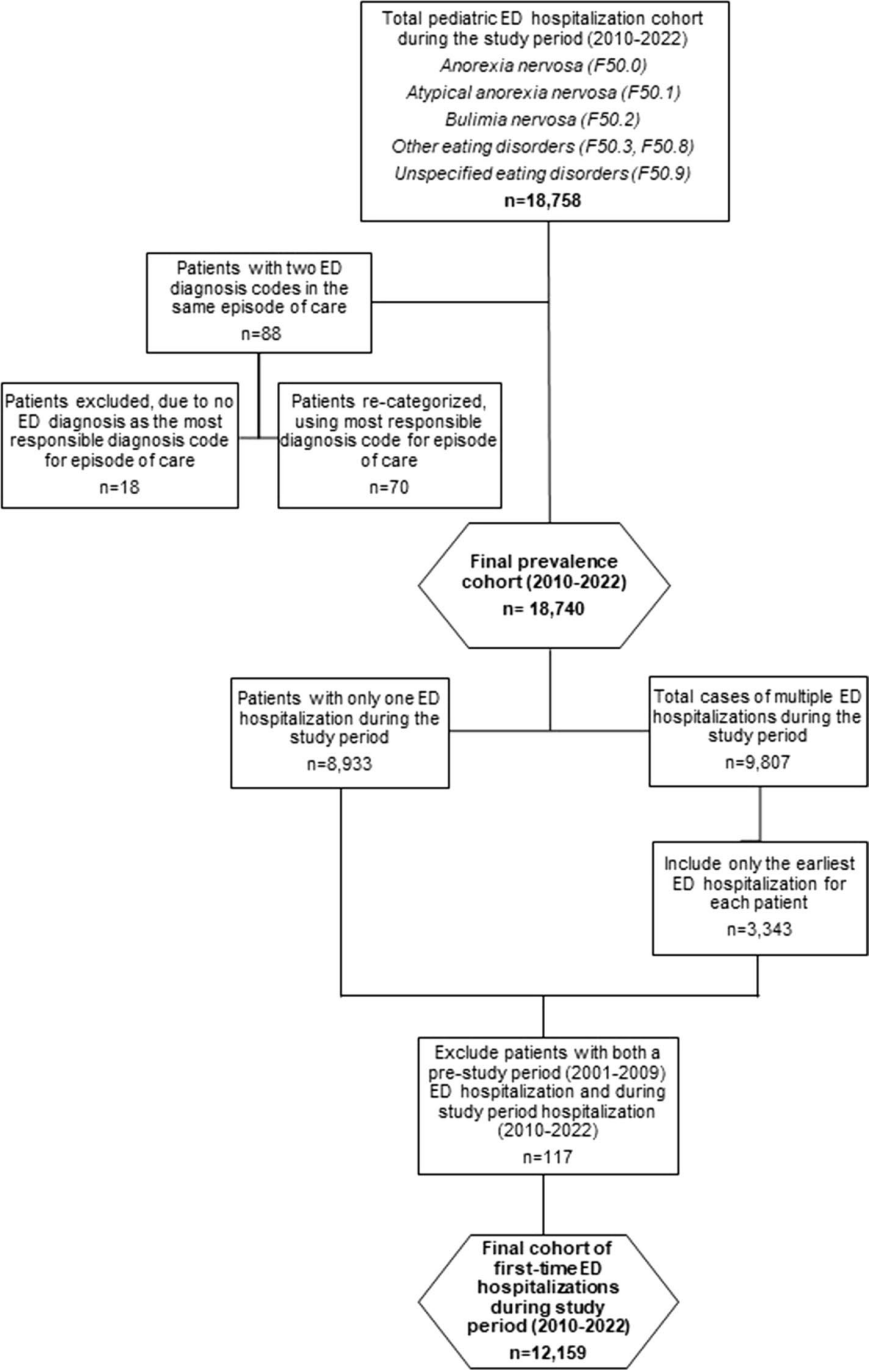
Process of pediatric eating disorder hospitalization case selection in the Discharge Abstract Database, using ICD-10 codes. Abbreviations: ICD-10, International Classification of Diseases, 10th Revision Notes: Pre-study period (2001–2009), study period (2010–2022)

Figure 1 summarizes the inclusion criteria of pediatric patients in our cohort. To identify ED hospitalizations during the study period, all 25 diagnostic fields in the DAD were searched from 2010 to 2022 using the selected ICD-10 codes. To minimize misclassification, patients with two or more ED diagnostic codes reported in a single episode of care (n = 88) were recategorized using the most responsible diagnostic code. In cases where this was not possible, patients were excluded (n = 18, 0.1%). Other ICD-10 codes related to disordered eating behaviours (F50.4, F50.5, F98.2, F98.3 and R63.0, R63.2 to R63.6) were not included in this study, as these codes are primarily used when diagnosing feeding disorders or indicating signs and symptoms concerning food intake.

Using the prevalence cohort, we identified first-time ED hospitalizations during the study period, using the patient’s scrambled health card number. A total of 8,933 patients had only one hospitalization during the study period and 3,343 patients had multiple hospitalizations. Among those with multiple hospitalizations, only the first documented hospitalization was included. Additionally, health card numbers were searched in a pre-study period (2001–2009) to ensure that patients were not hospitalized prior to our study period. Patients who also had an ED hospitalization in the pre-study period were not included as having a first-time hospitalization during the study period. The years 2001 to 2009 were selected as the pre-study period, as this was the earliest data available using ICD-10 coding. We analyzed first-time ED hospitalizations to compare how characteristics of first-time hospitalization differed from total hospitalizations, especially in-terms of length of stay, discharge disposition, age at hospitalization and ED diagnosis.

### Study population and time period

ED hospitalizations were searched over a 13-year period starting in the 2010/11 fiscal year and ending in the 2022/23 fiscal year, beginning on April 1st and ending on March 31st the following year. Only patients between the ages of 5 and 17 years, inclusive, at the time of discharge were included.

### Statistical analyses

Results of total and first-time ED hospitalizations were presented as rates, proportions, means and medians, by sex, age group, province/territory, discharge disposition, hospital length of stay and ED diagnosis. Age and sex-specific rates per 100,000 population were calculated, using Statistics Canada national population estimates (excl. QC), over the 13-year period (2010/11 to 2022/23). Age-standardized rates per 100,000 population were standardized to the 2011 Canadian population (excl. QC) by direct standardization. Annual trends were quantified using average annual percent change (AAPC). Age-standardized rates that have significantly changed over the study period, were identified by an AAPC that is significantly different from zero at the alpha = 0.05 level [28]. Linear regression was used to calculate the mean change in length of hospital stay and age at hospitalization during the study period.

SAS EG version 7.1 (SAS Institute Inc., Cary, NC, USA) was used to conduct all descriptive analyses. Joinpoint Regression Program version 5.0.2 (SEERStat, NCI, Bethesda, MD, USA) was used to conduct trend analyses. Cells with small counts between one and four as well as rates that would allow for calculation of small cells have been presented as a range, to reduce potential identification of individuals and comply with CIHI’s data sharing agreements.

## Results

Between 2010/11 to 2022/23, there were 18,740 total cases of ED hospitalizations among children and youth, of which, 12,159 (64.9%) patients had a first-time hospitalization for an ED. About half of the total (51.3%) and first-time (47.3%) ED hospitalization cases were diagnosed as AN. Females and males had similar distributions of ED diagnoses, however, males had a higher proportion of other EDs among both total (Male: 23.3% vs. Female: 8.7%) and first-time hospitalizations (Male: 25.5% vs. Female: 9.6%), compared to females (Additional file 1: Table A1).

**Table 1:** Age and sex-specific rates, per 100,000 population, total and first-time cases of ED hospitalizations among children and youth (5 to 17 years), Canada (excl. QC), 2010/11 to 2022/23

	Ages 5 to 10 years				Ages 11 to 17 years				Ages 5 to 17 years	
	Total cases		First-time cases		Total cases		First-time cases		Total cases	First-time cases
	Count	Rate (95% CI)	Count	Rate (95% CI)	Count	Rate (95% CI)	Count	Rate (95% CI)	Rate (95% CI)	Rate (95% CI)
Females	315	2.7 (2.4, 3.0)	255	2.2 (1.9, 2.5)	16 822	119.4 (117.6, 121.2)	10 718	76.1 (74.7, 77.5)	66.7 (65.7, 67.7)	42.7 (41.9, 43.5)
Anorexia nervosa	107	0.9 (0.7, 1.1)	83	0.7 (0.6, 0.9)	8 802	62.5 (61.2, 63.8)	5 198	36.9 (35.9, 37.9)	34.7 (34.0, 35.4)	20.6 (20.0, 21.1)
Atypical anorexia nervosa	5	0.0 (0.0, 0.1)	1–4	0.0 (0.0, 0.1)	453	3.2 (2.9, 3.5)	273	1.9 (1.7, 2.2)	1.8 (1.6, 1.9)	1.1 (1.0, 1.2)
Bulimia nervosa	9	0.1 (0.0, 0.1)	1–4	0.0 (0.0, 0.0)	1 565	11.1 (10.6, 11.7)	900	6.4 (6.0, 6.8)	6.1 (5.8, 6.4)	3.5 (3.3, 3.7)
Other	128	1.1 (0.9, 1.3)	110	0.9 (0.8, 1.1)	1 375	9.6 (9.1, 10.1)	943	6.7 (6.3, 7.1)	5.8 (5.5, 6.1)	4.1 (3.9, 4.3)
Unspecified	66	0.6 (0.4, 0.7)	57	0.5 (0.4, 0.6)	4 645	33.0 (32.0, 33.9)	3 404	24.2 (23.4, 25.0)	18.3 (17.8, 18.9)	13.5 (13.0, 13.9)
Males	145	1.2 (1.0, 1.4)	118	1.0 (0.8, 1.1)	1 441	9.7 (9.2, 10.2)	1 059	7.2 (6.7, 7.6)	5.9 (5.6, 6.2)	4.4 (4.1, 4.6)
Anorexia nervosa	34	0.3 (0.2, 0.4)	24	0.2 (0.1, 0.3)	669	4.5 (4.2, 4.9)	380	3.0 (2.7, 3.3)	2.6 (2.4, 2.8)	1.8 (1.6, 1.9)
Atypical anorexia nervosa	1–4	0.0 (0.0, 0.1)	1–4	0.0 (0.0, 0.0)	29	0.2 (0.1, 0.3)	25	0.2 (0.1, 0.2)	0.1 (0.1, 0.2)	0.1 (0.1. 0.1)
Bulimia nervosa	1–4	0.0 (0.0, 0.0)	1–4	0.0 (0.0, 0.0)	84	0.6 (0.4, 0.7)	57	0.4 (0.3, 0.5)	0.3 (0.2, 0.4)	0.2 (0.2, 0.3)
Other	82	0.7 (0.5, 0.8)	70	0.6 (0.4, 0.7)	287	1.9 (1.7, 2.2)	230	1.6 (1.4, 1.8)	1.4 (1.2, 1.5)	1.1 (1.0, 1.2)
Unspecified	26	0.2 (0.1, 0.3)	22	0.2 (0.1, 0.3)	372	2.5 (2.3, 2.8)	299	2.0 (1.8, 2.2)	1.5 (1.3, 1.6)	1.2 (1.1, 1.3)

Data source: Discharge Abstract Database 2010/2011–2022/23

Abbreviations: CI, confidence interval; ED, eating disorder

Notes: Age-specific rates per 100,000 are calculated using Statistics Canada national population estimates (excl. QC) from 2010 to 2022, with 95% CIs. Patients with a gender other than male or female have been excluded (n = 17) due to small sample sizes

Hospitalization data with small counts between one and four as well as rates that would allow for derivation of small counts have been presented as a range

Across all ED diagnoses, females had significantly higher rates of total (Female: 66.7/100,000 vs. Male: 5.9/100,000) and first-time (Female: 42.7/100,000 vs. Male: 4.4/100,000) ED hospitalizations compared to males (Table 1). Youth ages 11 to 17 years had significantly higher rates of ED hospitalizations compared to children ages 5 to 10 years.

**Fig. 2:**
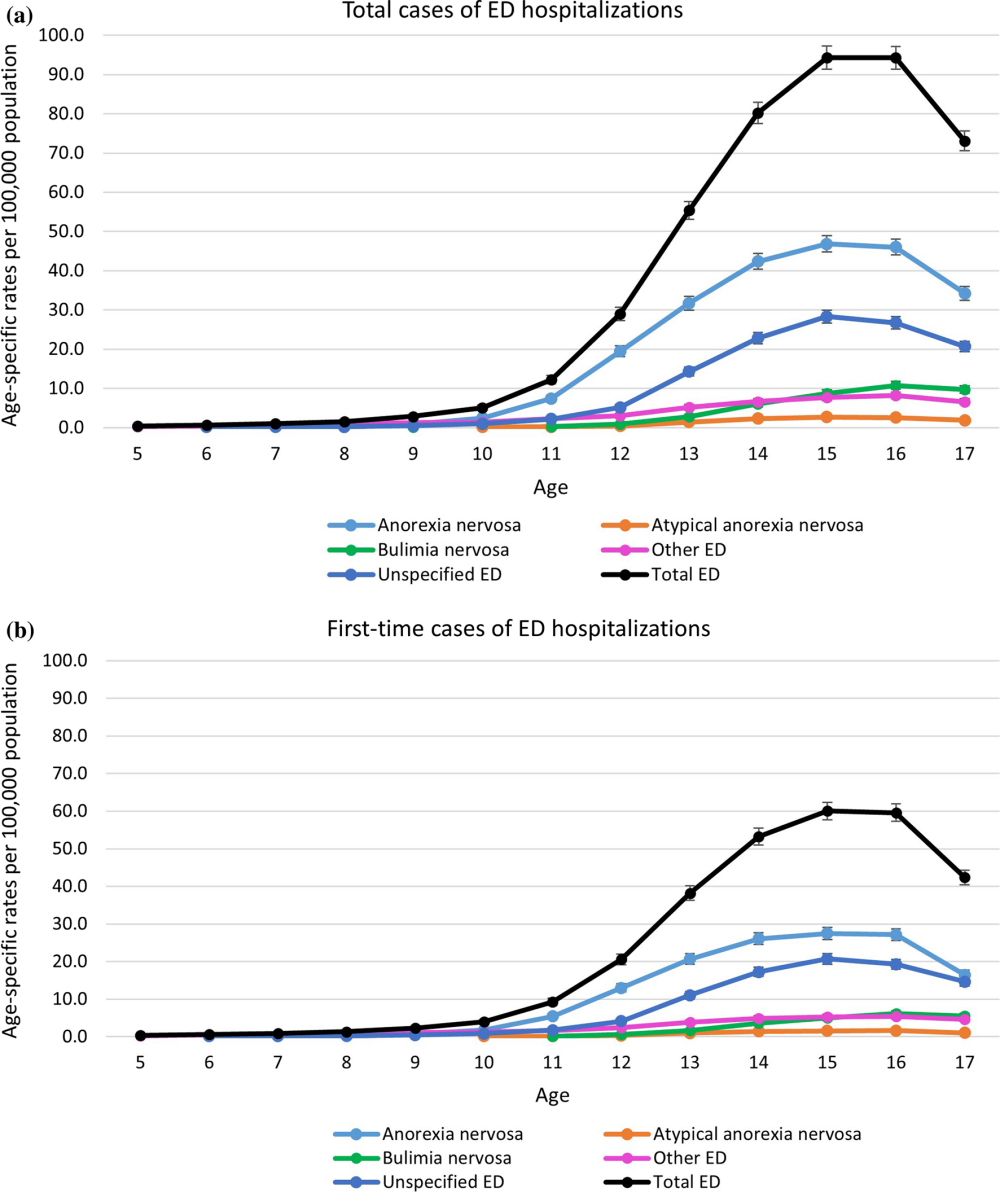
**a** and **b**. Age-specific rates of ED hospitalizations among children and youth, 2010/11 to 2022/23, by diagnosis *Data source*: Discharge Abstract Database 2010/2011–2022/23. Abbreviations: ED, eating disorder Hospitalization data with small counts between one and four as well as rates that would allow for derivation of small counts have been presented as a range

The average age of total ED hospitalizations among children and youth was 14.7 (95% CI: 14.7, 14.7) years and among first-time ED hospitalizations was 14.6 (95% CI: 14.5, 14.6) years. Youth aged 15 and 16 years had the highest rates of first-time and total ED hospitalizations, respectively. (Fig. 2 a & b). Males had significantly earlier average ages (years) of total [Male: 14.0 (95% CI: 13.9, 14.1) vs. Female: 14.8 (95% CI: 14.8, 14.8)] and first-time [Male: 13.9 (95% CI: 13.8, 14.0) vs. Female: 14.6 (95% CI: 14.6, 14.6)] ED hospitalizations. During the study period, the average age of ED hospitalizations significantly decreased, for total cases (2010: 14.8 years vs. 2022: 14.5 years, *p* < *0.05*) and first-time cases (2010: 14.7 years vs. 2022: 14.4 years, *p* < *0.05*).

**Fig. 3:**
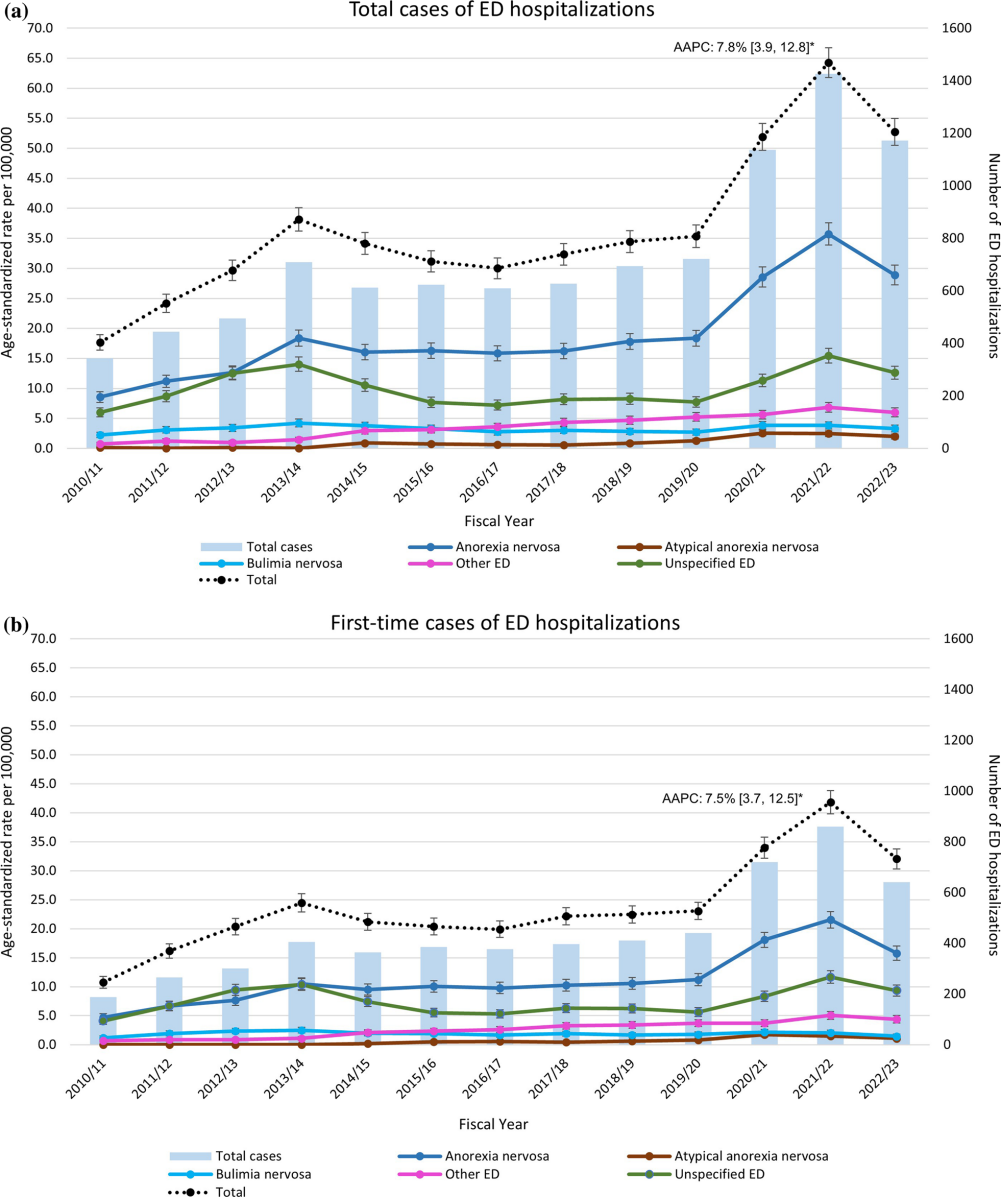
**a** and **b**. Age-standardized rates of total and first-time ED hospitalizations among children and youth, 2010/11 to 2022/23, by year and diagnosis* Data source*: Discharge Abstract Database 2010/2011–2022/23. Abbreviations: CI, confidence interval; AAPC, average annual percent change; ED, eating disorder. Notes: Age-standardized rates per 100,000 population are standardized to the 2011 Canadian population (excl. Quebec) using direct standardization

Between 2010/11 and 2022/23, there was an overall significant increase in total and first-time ED hospitalizations. Rates of total ED hospitalizations increased from 17.7 cases per 100,000 population in 2010/11 to 52.8 cases per 100,000 in 2022/23, this corresponds to an AAPC of 7.8% (3.9, 12.8) (Fig. 3a). Similarly, rates of first-time ED hospitalizations increased from 10.7 to 32.0 cases per 100,000, with an AAPC of 7.5% (3.7, 12.5) (Fig. 3b). Among total ED hospitalization cases, a relative increase of 108.0% occurred from 2010/11 to 2013/14, followed by relatively stable ED hospitalizations between 2013/14 and 2019/20. Between 2019/20 and 2021/22, cases increased again by 84.5%, followed by a decline of 16.5% in 2022/23. Although total ED hospitalization cases began to decline in 2022/23, they remained higher than pre-pandemic (2019/20) (Fig. 3a). Similar trends were observed among first-time ED hospitalization cases with a significant increase from 2010/11 to 2013/14 and from 2019/20 to 2021/22 (Fig. 3b). Although during the study period, there is year-to-year variation in rates of total and first-time ED hospitalizations, overall trends have increased across each ED diagnosis (Additional file 1: Table A2).

**Fig. 4:**
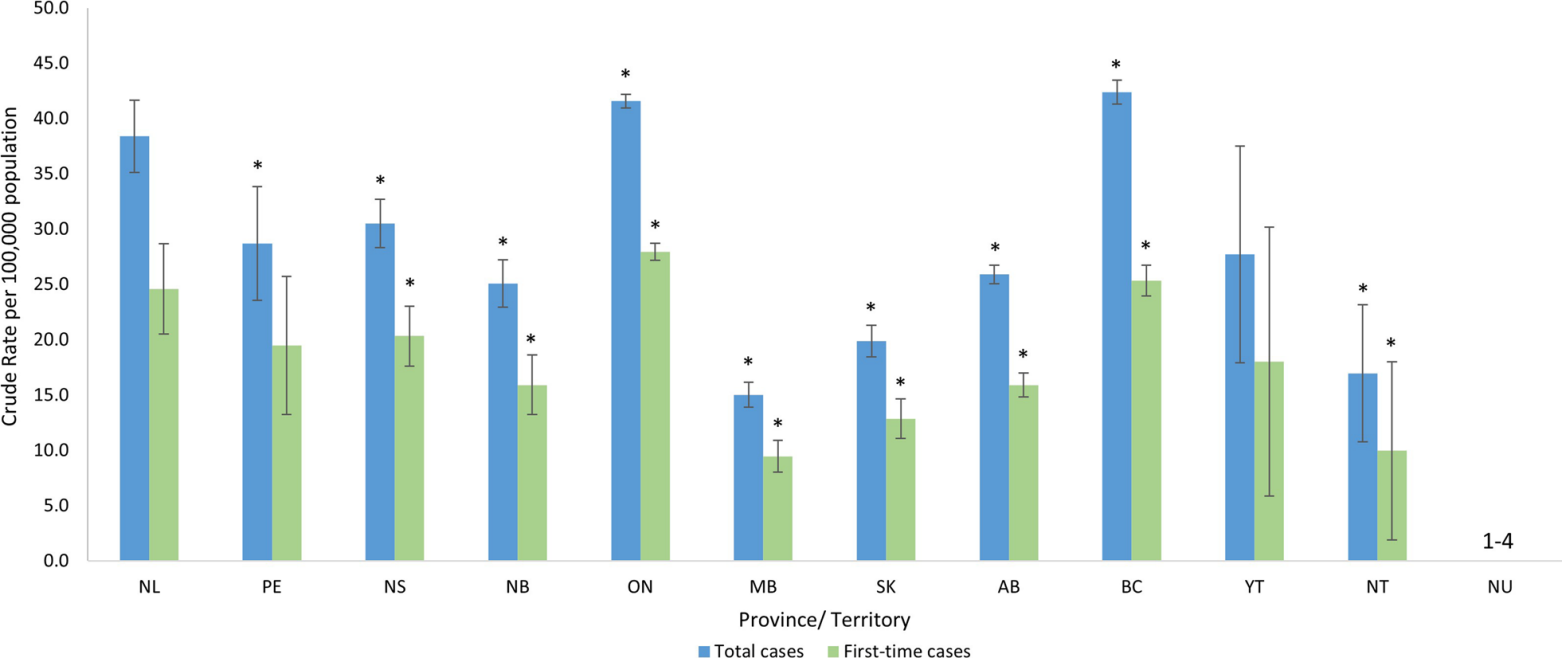
Crude rates of ED hospitalizations, 2010/11 to 2022/23, by provinces and territories *Data source*: Discharge Abstract Database 2010/2011–2022/23 Abbreviations: ED, eating disorder. Notes: The rate for each province/territory was compared to the national rate minus the province/territory being compared. *P < *0.05* Hospitalization data with small counts between one and four as well as rates that would allow for derivation of small counts have been presented as a range

Ontario﻿ and British Columbia had significantly higher rates of total and first-time ED-hospitalizations whereas Nova Scotia, New Brunswick, Manitoba, Saskatchewan, Alberta and Northwest Territories all had significantly lower rates, compared to the rest of Canada (Fig. 4).

**Table 2:** Length of hospital stay (days) of total and first-time ED hospitalizations among children and youth (5–17 years), Canada (excl. QC), 2010/11 to 2022/23

	Anorexia Nervosa	Atypical Anorexia Nervosa	Bulimia Nervosa	Other	Unspecified	Total
Total cases						
Mean (95% CI)	25.6 (25.0, 26.2)	16.5 (14.7, 18.3)	18.3 (16.4, 20.2)	18.0 (17.0, 19.0)	13.1 (12.6, 13.6)	20.6 (20.2, 21.0)
Median [IQR]	16.0 [7.0, 33.0]	10.0 [5.0, 20.0]	10.0 [4.0, 20.0]	11.0 [5.0, 21.0]	7.0 [3.0, 16.0]	12.0 [5.0, 25.0]
Mean difference from 2010 to 2022 (95% CI)	− 17.8 (− 21.2, − 14.4)*	− 43.6 (− 59.8, − 27.4)*	− 19.6 (− 28.1, − 11.1)*	− 9.9 (− 15.8, − 4.0)*	− 13.8 (− 17.5, − 10.1)*	− 15.9 (− 18.2, − 13.6)*
Annual change (95% CI)	− 1.5 (− 1.7, − 1.3)*	− 1.7 (− 2.3, − 1.1)*	− 1.4 (− 1.9, − 0.9)*	− 1.1 (− 1.4, − 0.8)*	− 0.8 (− 0.9, − 0.7)*	− 1.1 (− 1.2, − 1.0)*
First-time cases						
Mean (95% CI)	24.6 (24.0, 25.2)	14.7 (13.2, 16.2)	15.8 (14.5, 17.1)	17.1 (16.0, 18.2)	12.6 (12.0, 13.2)	19.1 (18.7, 19.5)
Median [IQR]	17.0 [9.0, 31.0]	11.0 [6.0, 20.0]	9.0 [4.0, 19.0]	11.0 [5.0, 21.0]	7.0 [3.0, 15.0]	12.0 [6.0, 24.0]
Mean difference from 2010–2022 (95% CI)	− 18.3 (− 21.8, − 14.8)*	− 25.2 (− 40.4, − 10.0)*	− 9.3 (− 17.4, -1.2)*	− 10.2 (− 16.1, -4.4)*	− 13.2 (− 18.0, − 8.4)*	− 14.6 (− 17.1, − 12.1)*
Annual change (95% CI)	− 1.5 (− 1.7, − 1.3)*	− 1.4 (− 2.0, − 0.8)*	− 1.0 (− 1.3, − 0.7)*	− 1.1 (− 1.4, − 0.8)*	− 0.7 (− 0.9, − 0.5)*	− 1.0 (− 1.1, − 0.9)*

Data source: Discharge Abstract Database 2010/2011–2022/23

Abbreviations: CI, confidence interval; IQR, interquartile range; ED, eating disorder

^*^P < *0.05*

Among cases of total and first-time ED hospitalizations, those diagnosed with AN had a significantly longer average length of stay (LOS) compared to all other EDs examined (Table 2).

From 2010 to 2022, the LOS for total and first-time ED hospitalizations significantly declined. Patients hospitalized for an ED were most frequently discharged home, either with or without support services (Table 3). Fewer patients were transferred to other inpatient hospital care, left against medical advice, were transferred to continuing care or died while in care.

**Table 3:** Discharge disposition of total and first-time ED hospitalizations among children and youth (5–17 years), Canada (excl. QC), 2010/11 to 2022/23

	Anorexia Nervosa	Atypical Anorexia Nervosa	Bulimia Nervosa	Other	Unspecified	Total
	N (%)					
Total cases						
Discharged home^ƚ^	8 554 (88.9)	443 (90.4)	1 471 (88.7)	1 696 (91.3)	4 509 (88.1)	16 673 (89.0)
Transferred to inpatient hospital care^ǂ^	729 (7.6)	27 (5.5)	113 (6.8)	90 (4.8)	365 (7.1)	1 324 (7.1)
Left against medical advice	198 (2.1)	8 (1.6)	34 (2.0)	29 (1.6)	100 (2.0)	369 (2.0)
Transferred to continuing care^§^	103 (1.1)	9 (1.8)	41 (2.5)	42 (2.3)	107 (2.1)	331 (1.8)
Emergency department or ambulatory care^¶^	11 (0.1)	1–4 (< 5.0)	0 (0.0)	1–4 (< 5.0)	1–4 (< 5.0)	12 (0.1)
Other^#^	22 (0.2)	1–4 (< 5.0)	0 (0.0)	1–4 (< 5.0)	34 (0.7)	31 (0.2)
First-time cases						
Discharged home^ƚ^	5 248 (91.2)	284 (93.4)	871 (90.8)	1 252 (92.5)	3 382 (89.3)	11 037 (90.8)
Transferred to inpatient hospital care^ǂ^	345 (6.0)	8 (2.6)	50 (5.2)	57 (4.2)	226 (6.0)	686 (5.6)
Left against medical advice	106 (1.8)	7 (2.3)	22 (2.3)	25 (1.8)	89 (2.3)	249 (2.0)
Transferred to continuing care^§^	32 (0.6)	1–4 (< 5.0)	15–19 (< 5.0)	20 (1.5)	86 (2.3)	156 (1.3)
Emergency department or ambulatory care^¶^	5–9 (< 5.0)	0 (0.0)	0 (0.0)	0 (0.0)	1–4 (< 5.0)	8 (0.1)
Other^#^	16 (0.3)	1–4 (< 5.0)	0 (0.0)	0 (0.0)	1–4 (< 5.0)	23 (0.2)

Data source: Discharge Abstract Database 2010/2011–2022/23

Abbreviations: ED, Eating disorder

^ƚ^ Discharged to a private home with supports from the community at home or referred to services, or discharged to a private home without any supports or referrals

^ǂ^ Includes patients transferred to other acute care within the current facility or to another facility

^§^Includes forms of continuing care such as long-term care facility, palliative care, residential or group/support living or addiction treatment centre where medical care may continue to be provided by medical staff

^¶^ Includes transfers to reporting facility or another facility for emergency or ambulatory care

^#^ Includes those who died (n < 5) and those who did not return from pass or leave

Notes: Hospitalization data with small counts between one and four as well as percentages that would allow for derivation of small counts have been presented as a range

## Discussion

In Canada between 2010/11 and 2022/23 there were 18,740 ED hospitalizations among children and youth, with 64.9% being first-time hospitalizations. The majority of ED hospitalizations in Canada were among females and youth. The rates of total and first-time ED hospitalizations significantly increased during the study period, with the highest rates reported in 2021. Across Canada, Ontario and British Columbia had the highest rates of ED hospitalizations.

Rates of ED hospitalizations were significantly higher among females compared to males, across all ages and diagnoses, in our study. Higher rates of EDs among females is well documented in the literature, however, there are still a considerable number of males with EDs [6, 8, 9, 11, 12, 15, 20, 29]. The distribution of ED diagnoses was similar between males and females in our study, however, males had a higher proportion of other EDs compared to females. This difference may be due to males not meeting the diagnostic criteria or having different clinical presentations for EDs, and therefore are being captured in the other ED category [9, 29, 30].Additionally, males are more likely than females to present with ARFID [29–33], which falls into our category of other EDs.

Youth had significantly higher rates of ED hospitalizations compared to children in our study. Throughout adolescence the rates of ED hospitalizations increased, with a peak at ages 15 and 16 years. The age at onset for most EDs occurs during adolescence (ages 14 to 18) [2, 8, 13, 15, 34], however, several studies have reported that the age of onset for many EDs has decreased over the past decade, with more children under the age of 10 years being diagnosed [2, 8, 13–15]. In our study, the mean age for an ED hospitalization significantly decreased from 2010 to 2022, with the lowest average ages seen during the pandemic period. There are many factors that may influence the shifting age at hospitalization, including changes to cultural or personal norms, earlier access to social media, and participation in weight or body-image centered sports/activities [2, 3, 8, 14]. During the pandemic, children spent more time at home, which may have led to caregivers being more aware of the child’s eating behaviours and cognition, potentially resulting in earlier encounters with the healthcare system [21, 35]. Males in our study were hospitalized for an ED at significantly younger ages than females. Some research confirms this finding [11, 15], whereas other studies report no clear sex-differences of age of onset [7, 31]. Additionally, males are more frequently diagnosed with ARFID, which is an ED that is typically diagnosed earlier than AN or BN, [29–33] which may explain the younger age of hospitalization among males in our study.

Our trend analysis showed an initial increase in ED hospitalizations from 2010/11 to 2013/14 after which, trends remained stable until 2019/20, followed by a spike from 2020/21 to 2022/23. In 2013, the Diagnostic and Statistical Manual of Mental Disorders (DSM), was updated, with several changes made to the ED diagnostic criteria [36]. Although ICD-10 codes did not change during this time, physicians base their diagnoses on the criteria in the DSM-5. The new, broader criteria in the DSM-5 may have lead to more patients being diagnosed with AN and fewer patients being diagnosed with unspecified EDs, around that time [36, 37]. Several studies highlight the increase in ED cases during the pandemic period [18–21, 35, 38–40]. Results have shown that hospitalization rates for EDs in 2022 started to decline, but are still higher than pre-pandemic [21, 38, 41]. This trend may be due to public health measures such as lockdowns and school closures causing increased isolation [17, 18], as well as longer wait times or changes to treatment during the pandemic [17]. Additionally, it is reported that during the pandemic, there was an increased preoccupation with fitness, weight gain and social media, which may have contributed to new cases of disordered eating, as well as worsening symptoms among those already diagnosed [42]. These results, emphasize the need for early screening and monitoring of children and youth presenting with disordered eating behaviours, in order to implement early and consistent treatment, which may aid in minimizing cases of inpatient hospitalization for EDs.

Across provinces/territories, Ontario and British Columbia had significantly higher rates of both total and first-time ED hospitalizations. There is limited Canadian research examining ED epidemiology at the provincial/territorial level, however one recent study, of five provinces, noted that hospitalizations for EDs were higher in hospitals in Ontario, Quebec and British Columbia compared to hospitals in Alberta and Newfoundland and Labrador [18]. Inequitable access to specialized hospital-based ED units across Canadian provinces/territories may in part explain hospitalization patterns in British Columbia and Ontario, where there is greater accessibility to specialized ED units compared to the rest of the country (excluding Quebec) [43].

Patients hospitalized for AN had significantly longer average LOS compared to those hospitalized for other EDs. We found that from 2010 to 2022, the average LOS for patients with an ED decreased significantly. Among the limited research available on hospital LOS, results vary from about 12 days to over 200 days, [44–46] with LOS decreasing over the last several years [44]. Several factors such as presence of comorbid conditions, severity of the ED, hospital resources, access to outpatient treatments and the COVID-19 pandemic may have an influence on LOS [44, 45]. The majority of patients in our study were discharged home following hospitalization. One study found that more than half of patients were discharged to a medical practitioner, and about 20% were referred to outpatient care. [47]

### Limitations

This study has several limitations inherent to administrative data. The DAD is unable to report sociodemographic characteristics or gender, which would have strengthened our study as certain population groups are disproportionately affected by EDs [17, 48]. Results from Quebec could not be reported due to data sharing agreements. Lastly, our results may be biased towards more severe ED cases, as we only captured acute inpatient hospitalizations.

There may be some misclassification of first-time ED hospitalizations. Scrambled health card numbers were used to identify ED hospitalizations across Canada between 2001 and 2022. This method creates challenges in identifying patients who may have received a new health card number during this time.

Additionally, we were unable to present disaggregated results for ARFID and binge eating disorder, due to the limited ICD-10 codes for EDs. The codes available do not capture the full spectrum of ED presentations and several diagnoses are grouped together.

## Conclusion

In Canada, between 2010 and 2022, hospitalizations for EDs in the pediatric population increased significantly, with the highest rates reported in 2021. Females and youth comprised the majority of ED hospitalizations. Currently limited research is available, at a national level, examining long-term trends. These results highlight the need for early interventions and screening as well as resource reallocation and preparedness for future pandemics. Further work will explore types of co-occurring diagnoses with EDs, as well as examine how trends are evolving in the post-pandemic era.

### Supplementary Information

**Additional file 1**. Proportion of ED hospitalizations, by sex and diagnosis. 

**Additional file 2**. Age-standardized rates of ED hospitalizations, by year and diagnosis.

## Data Availability

Access to programming code and aggregated data can be made available upon request through the corresponding author.
