# Supplementary material for: Eating disorder hospitalizations among children and youth in Canada from 2010 to 2022: a population-based surveillance study using administrative data
Source: J Eat Disord. 2024 Jan 2;12:3. doi: 10.1186/s40337-023-00957-y (PMC10763198; doi:10.1186/s40337-023-00957-y)
Supplement: Supplementary file 1 — Additional file 1. Proportion of ED hospitalizations, by sex and diagnosis. [file 40337_2023_957_MOESM1_ESM.docx]

| Proportion of ED hospitalizations among children and youth, 2010/11 to 2022/23, by sex and diagnosis | | | | | | | |
| --- | --- | --- | --- | --- | --- | --- | --- |
| **Eating disorder diagnosis** | **ICD-10** | **Total cases** | | | **First-time cases** | | |
|  |  | Both sexes | Female | Male | Both sexes | Female | Male |
|  |  | N (%) | | | | | |
| **Total** |  | 18740 | 17137 | 1586 | 12159 | 10973 | 1177 |
| Anorexia nervosa | F50.0 | 9617 (51.3) | 8909 (52.0) | 703 (44.3) | 5754 (47.3) | 5281 (48.1) | 472 (40.1) |
| Atypical anorexia nervosa | F50.1 | 490 (2.6) | 458 (2.7) | 32 (2.0) | 304 (2.5) | 277 (2.5) | 27 (2.3) |
| Bulimia nervosa | F50.2 | 1659 (8.9) | 1574 (9.2) | 84 (5.3) | 959 (7.9) | 901 (8.2) | 57 (4.8) |
| Other | F50.3, F50.8 | 1858 (9.9) | 1485 (8.7) | 369 (23.3) | 1354 (11.1) | 1053 (9.6) | 300 (25.5) |
| Unspecified | F50.9 | 5116 (27.3) | 4711 (27.5) | 398 (25.1) | 3788 (31.2) | 3461 (31.5) | 321 (27.3) |

**Additional file 1:**

**Data source:** Discharge Abstract Database 2010/2011-2022/23

**Abbreviations:** ICD-10, International Classification of Diseases 10^th^ revision; ED, eating disorder
