# Supplementary material for: Eating disorder hospitalizations among children and youth in Canada from 2010 to 2022: a population-based surveillance study using administrative data
Source: J Eat Disord. 2024 Jan 2;12:3. doi: 10.1186/s40337-023-00957-y (PMC10763198; doi:10.1186/s40337-023-00957-y)
Supplement: Supplementary file 2 — Additional file 2. Age-standardized rates of ED hospitalizations, by year and diagnosis. [file 40337_2023_957_MOESM2_ESM.docx]

**Additional file 2:**

| Age-standardized rates of ED hospitalizations among children and youth, by year and diagnosis, 2010/11 to 2022/23 | | | | | | | | | | | | |
| --- | --- | --- | --- | --- | --- | --- | --- | --- | --- | --- | --- | --- |
|  | **Anorexia nervosa** | | **Atypical anorexia nervosa** | | **Bulimia nervosa** | | **Other** | | **Unspecified** | | **Total** | |
| **Total cases** | | | | | | | | | | | | |
| AAPC | 9.8% (6.4, 14.5)* | | 24.0% (16.4, 41.4)* | | 3.8% (2.2, 6.4)* | | 20.5% (15.3, 32.4)* | | 6.8% (4.3, 10.8)* | | 7.8% (3.9, 12.8)* | |
| **Year** | **Count** | **ASR (95% CI)** | **Count** | **ASR (95% CI)** | **Count** | **ASR (95% CI)** | **Count** | **ASR (95% CI)** | **Count** | **ASR (95% CI)** | **Count** | **ASR (95% CI)** |
| 2010/11 | 344 | 8.6 (7.7, 9.5) | 5 | 0.1 (0.0, 0.2) | 89 | 2.2 (1.8, 2.7) | 30 | 0.8 (0.5, 1.0) | 241 | 6.0 (5.2, 6.8) | 709 | 17.7 (16.4, 18.9) |
| 2011/12 | 445 | 11.2 (10.1, 12.2) | <5 | 0.0 (0.0, 0.3) | 123 | 3.1 (2.5, 3.6) | 45-49 | 1.2 (0.9, 1.6) | 346 | 8.7 (7.8, 9.6) | 964 | 24.2 (22.7, 25.7) |
| 2012/13 | 495 | 12.6 (11.5, 13.7) | 6 | 0.2 (0.0, 0.3) | 134 | 3.4 (2.8, 4.0) | 39 | 1.0 (0.7, 1.3) | 490 | 12.5 (11.4, 13.6) | 1164 | 29.7 (28.0, 31.4) |
| 2013/14 | 709 | 18.4 (17.0, 19.7) | <5 | 0.1 (0.0, 0.2) | 163 | 4.2 (3.6, 4.9) | 55-59 | 1.5 (1.1, 1.8) | 542 | 14.0 (12.9, 15.2) | 1475 | 38.2 (36.2, 40.1) |
| 2014/15 | 613 | 16.0 (14.8, 17.3) | 34 | 0.9 (0.6, 1.2) | 144 | 3.8 (3.2, 4.4) | 113 | 2.9 (2.4, 3.5) | 403 | 10.5 (9.5, 11.6) | 1307 | 34.2 (32.3, 36.0) |
| 2015/16 | 624 | 16.3 (15.0, 17.6) | 29 | 0.8 (0.5, 1.0) | 126 | 3.3 (2.7, 3.9) | 120 | 3.1 (2.6, 3.7) | 294 | 7.7 (6.8, 8.6) | 1193 | 31.1 (29.4, 32.9) |
| 2016/17 | 610 | 15.8 (14.6, 17.1) | 24 | 0.6 (0.4, 0.9) | 106 | 2.8 (2.2, 3.3) | 139 | 3.6 (3.0, 4.2) | 278 | 7.2 (6.4, 8.1) | 1157 | 30.0 (28.3, 31.7) |
| 2017/18 | 627 | 16.2 (14.9, 17.5) | 23 | 0.6 (0.4, 0.8) | 117 | 3.0 (2.5, 3.6) | 170 | 4.4 (3.7, 5.0) | 316 | 8.2 (7.3, 9.1) | 1253 | 32.3 (30.5, 34.1) |
| 2018/19 | 694 | 17.8 (16.5, 19.1) | 33 | 0.8 (0.5, 1.1) | 110 | 2.8 (2.3, 3.4) | 185 | 4.7 (4.0, 5.4) | 322 | 8.3 (7.4, 9.2) | 1344 | 34.4 (32.6, 36.3) |
| 2019/20 | 721 | 18.3 (17.0, 19.7) | 51 | 1.3 (0.9, 1.7) | 107 | 2.7 (2.2, 3.2) | 208 | 5.2 (4.5, 5.9) | 305 | 7.7 (6.9, 8.6) | 1392 | 35.3 (33.5, 37.2) |
| 2020/21 | 1137 | 28.6 (26.9, 30.2) | 102 | 2.6 (2.1, 3.1) | 153 | 3.8 (3.2, 4.4) | 226 | 5.6 (4.9, 6.4) | 451 | 11.3 (10.3, 12.4) | 2069 | 51.9 (49.7, 54.1) |
| 2021/22 | 1426 | 35.7 (33.8, 37.6) | 98 | 2.5 (2.0, 3.0) | 153 | 3.8 (3.2, 4.4) | 275 | 6.8 (6.0, 7.6) | 616 | 15.4 (14.2, 16.6) | 2568 | 64.3 (61.8, 66.7) |
| 2022/23 | 1172 | 28.9 (27.2, 30.5) | 80 | 2.0 (1.5, 2.4) | 134 | 3.3 (2.7, 3.9) | 247 | 6.0 (5.3, 6.8) | 512 | 12.6 (11.5, 13.7) | 2145 | 52.8 (50.5, 55.0) |
| **First-time cases** | | | | | | | | | | | | |
| AAPC | 9.9% (5.2, 16.6)* | | 28.0% (22.2, 74.0)* | | 2.2% (-4.3, 7.1) | | 19.0% (15.8, 25.8)* | | 8.2% (5.1, 13.2)* | | 7.5% (3.7, 12.5)* | |
| **Year** | **Count** | **ASR (95% CI)** | **Count** | **ASR (95% CI)** | **Count** | **ASR (95% CI)** | **Count** | **ASR (95% CI)** | **Count** | **ASR (95% CI)** | **Count** | **ASR (95% CI)** |
| 2010/11 | 188 | 4.7 (4.0, 5.3) | <5 | 0.1 (0.0, 0.1) | 48 | 1.2 (0.9, 1.5) | 25-29 | 0.7 (0.4, 1.0) | 164 | 4.1 (3.5, 4.7) | 431 | 10.7 (9.7, 11.7) |
| 2011/12 | 265 | 6.7 (5.8, 7.5) | <5 | 0.0 (0.0, 0.1) | 77 | 1.9 (1.5, 2.4) | 35-39 | 0.9 (0.6, 1.2) | 266 | 6.7 (5.9, 7.5) | 644 | 16.2 (14.9, 17.4) |
| 2012/13 | 300 | 7.7 (6.8, 8.5) | <5 | 0.1 (0.0, 0.2) | 92 | 2.4 (1.9, 2.8) | 30-34 | 0.9 (0.6, 1.2) | 370 | 9.4 (8.5, 10.4) | 799 | 20.4 (19.0, 21.8) |
| 2013/14 | 405 | 10.5 (9.5, 11.5) | <5 | 0.1 (0.0, 0.2) | 96 | 2.5 (2.0, 3.0) | 40-44 | 1.1 (0.7, 1.4) | 400 | 10.4 (9.3, 11.4) | 946 | 24.5 (22.9, 26.0) |
| 2014/15 | 364 | 9.5 (8.5, 10.5) | 7 | 0.2 (0.0, 0.3) | 75 | 2.0 (1.5, 2.4) | 82 | 2.1 (1.7, 2.6) | 284 | 7.4 (6.6, 8.3) | 812 | 21.2 (19.8, 22.7) |
| 2015/16 | 385 | 10.1 (9.1, 11.0) | 20 | 0.5 (0.3, 0.8) | 74 | 1.9 (1.5, 2.4) | 91 | 2.4 (1.9, 2.8) | 212 | 5.5 (4.8, 6.3) | 782 | 20.4 (19.0, 21.8) |
| 2016/17 | 377 | 9.8 (8.8, 10.8) | 21 | 0.6 (0.3, 0.8) | 64 | 1.7 (1.2, 2.1) | 101 | 2.6 (2.1, 3.1) | 205 | 5.3 (4.6, 6.0) | 768 | 19.9 (18.5, 21.3) |
| 2017/18 | 397 | 10.3 (9.3, 11.2) | 18 | 0.5 (0.2, 0.7) | 73 | 1.9 (1.5, 2.3) | 127 | 3.3 (2.7, 3.8) | 244 | 6.3 (5.5, 7.1) | 859 | 22.2 (20.7, 23.6) |
| 2018/19 | 411 | 10.5 (9.5, 11.6) | 24 | 0.6 (0.4, 0.8) | 64 | 1.7 (1.2, 2.1) | 134 | 3.4 (2.8, 4.0) | 244 | 6.3 (5.5, 7.0) | 877 | 22.5 (21.0, 23.9) |
| 2019/20 | 441 | 11.2 (10.2, 12.2) | 31 | 0.8 (0.5, 1.1) | 69 | 1.8 (1.3, 2.2) | 147 | 3.7 (3.1, 4.3) | 222 | 5.6 (4.9, 6.4) | 910 | 23.1 (21.6, 24.6) |
| 2020/21 | 720 | 18.1 (16.8, 19.4) | 69 | 1.7 (1.3, 2.1) | 86 | 2.2 (1.7, 2.6) | 148 | 3.7 (3.1, 4.3) | 332 | 8.3 (7.4, 9.2) | 1355 | 34.0 (32.2, 35.8) |
| 2021/22 | 860 | 21.5 (20.1, 23.0) | 60 | 1.5 (1.1, 1.9) | 82 | 2.1 (1.6, 2.5) | 204 | 5.1 (4.4, 5.7) | 466 | 11.7 (10.6, 12.7) | 1672 | 41.8 (39.8, 43.8) |
| 2022/23 | 641 | 15.8 (14.6, 17.0) | 44 | 1.1 (0.8, 1.4) | 59 | 1.5 (1.1, 1.8) | 181 | 4.4 (3.8, 5.0) | 379 | 9.3 (8.4, 10.3) | 1304 | 32.0 (30.3, 33.8) |

**Data source:** Discharge Abstract Database 2010/2011-2022/23

**Abbreviations:** CI, confidence interval; AAPC, average annual percent change

**Notes:** Age-standardized rates per 100,000 population are standardized to the 2011 Canadian population (excl. QC) using direct standardization, with 95% CIs

**P <0.05*

Hospitalization data with small counts between one and four as well as rates that would allow for derivation of small counts have been presented as a range.
